# Emotional Eating and Cardiovascular Risk Factors in the Police Force: The Carolina Blue Project

**DOI:** 10.3390/ijerph21030332

**Published:** 2024-03-12

**Authors:** Ya-Ke Wu, Tany G. Pacchioni, Anil K. Gehi, Katherine E. Fitzgerald, Divya V. Tailor

**Affiliations:** 1 School of Nursing, University of North Carolina at Chapel Hill, Chapel Hill, NC 27599, USA; 2 Department of Psychiatry, University of North Carolina at Chapel Hill, Chapel Hill, NC 27599, USA; 3 Department of Psychology and Neuroscience, University of North Carolina at Chapel Hill, Chapel Hill, NC 27599, USA; tanypacc@unc.edu; 4 Division of Cardiology, University of North Carolina at Chapel Hill, Chapel Hill, NC 27599, USA; anil_gehi@med.unc.edu; 5 Department of Biology, University of North Carolina at Chapel Hill, Chapel Hill, NC 27599, USA; krazykat@email.unc.edu; 6 Department of Biostatistics, University of North Carolina at Chapel Hill, Chapel Hill, NC 27599, USA; dvtailor@unc.edu

**Keywords:** emotional eating, cardiovascular disease risk factors, law enforcement officers, police force, North Carolina, the Carolina Blue Project

## Abstract

There is an association between emotional eating and cardiovascular disease (CVD) risk factors; however, little is known about this association in the police force. This study explores the associations between emotional eating and CVD risk factors in law enforcement officers in North Carolina. Four hundred and five officers completed The Emotional Eating Scale, and 221 of them completed the assessment for CVD-related markers. Descriptive statistics, Pearson’s correlation, and multiple linear regression analyses were performed. Emotional eating in response to anger was significantly positively associated with body weight (*β* = 1.51, *t* = 2.07, *p* = 0.04), diastolic blood pressure (*β* = 0.83, *t* = 2.18, *p* = 0.03), and mean arterial pressure (*β* = 0.84, *t* = 2.19, *p* = 0.03) after adjusting for age and use of blood pressure medicine. Emotional eating in response to depression was significantly positively associated with triglycerides (*β* = 5.28, *t* = 2.49, *p* = 0.02), while the emotional eating in response to anxiety was significantly negatively associated with triglycerides (*β* = −11.42, *t* = −2.64, *p* = 0.01), after adjusting for age and use of cholesterol medicine. Our findings offer new insights to address emotional eating and lower CVD risk in law enforcement officers.

## 1. Introduction

Cardiovascular disease (CVD) remains the most significant cause of mortality in the United States, accounting for 695,000 deaths in 2021 [1]. CVD encompasses a range of disorders affecting the heart and blood vessels, including coronary artery disease, cerebrovascular disease, and hypertension, each contributing to this staggering mortality rate [2]. Law enforcement officers in the United States have the highest CVD mortality rate of all occupations and are particularly vulnerable to CVD, driven by unique occupational stressors and lifestyle challenges [3,4]. Studies have identified a higher prevalence of CVD risk factors in law enforcement officers compared to the general public, including increased rates of obesity, hypertension, and metabolic syndrome [5].

The implications of CVD risk factors in law enforcement officers are far-reaching, impacting not only their personal health but also their professional capabilities and public safety responsibilities [6]. The physical and mental readiness of law enforcement officers is critical for their effective performance in high-stakes situations, with their health directly impacting their ability to respond effectively [7]. Notably, officers with CVD or its risk factors are at an increased risk of on-duty sudden cardiac events, a leading cause of mortality among active-duty law enforcement officers [8]. Nationally, a study of police officers in the Buffalo Cardio-Metabolic Occupational Police Stress study highlighted the significant disparity in CVD risk factors between law enforcement officers and the general employed population [3]. For example, a much higher percentage of officers were found to be obese (40.5% vs. 32.1% in the general population), have metabolic syndrome (26.7% vs. 18.7%), and have higher mean serum total cholesterol levels (200.8 mg/dL vs. 193.2 mg/dL) [3].

In North Carolina (NC), the situation may be exacerbated, as the risk of CVD death (170.9 per 100,000 in 2021) and risk of CVD event (9.8% risk of myocardial infarction or stroke) surpasses the national average (9.1% risk of myocardial infarction or stroke) [9,10]. Unfortunately, the prevalence of CVD risk factors among law enforcement officers in NC is unknown. The most recent study assessing law enforcement officers’ cardiovascular health in NC focused on the impact of air pollution on heart rhythm and non-CVD-related blood biomarkers, such as blood cell counts, but did not explore CVD risk factors in the context of emotional eating behaviors, which is an emerging area of concern [11].

Emotional eating refers to the tendency (or the urgency) of individuals to eat in response to emotions such as stress, sadness, boredom, or anger rather than in response to physical hunger [12]. While not officially recognized as a mental disorder in the Diagnostic and Statistical Manual of Mental Disorders, Fifth Edition [13], studies have shown connections between emotional eating and risk factors for CVD [14,15]. For example, more emotional eating was associated with higher body mass index (BMI) in 600 Italian young adults [16]. A longitudinal study suggested that emotional eating was linked to decreased high-density lipoprotein cholesterol (HDL) levels in Korean men and women (*n* = 1876) [17]. Young adults with higher degrees of emotional eating had a greater incidence of metabolic syndrome in a cross-sectional study (*n* = 104) [18]. Several studies have evaluated emotional eating among military personnel in the United States (U.S.) [19,20,21]. Emotional eating was associated with higher BMI and lower aerobic activity among U.S. Army and other active duty military service members [19,21]. However, research in military personnel did not explore emotional eating’s impact on CVD risk factors beyond BMI. In addition, prior research on military personnel has measured emotional eating using four different scales: the Motivation for Eating Scale [22], the Intuitive Eating Scale [23], the Three-factor Eating Questionnaire [24], and the Dutch Eating Behavior Questionnaire [25]. However, these scales do not specifically measure the tendency to eat in response to different types of emotion, such as anger, depression, or anxiety, which is measured by the Emotional Eating Scale [26].

To our knowledge, no study has investigated the relationship between emotional eating and CVD risk factors among NC law enforcement officers. The majority of research on law enforcement officers’ eating has focused on dietary composition (i.e., caloric consumption and percentage of dietary fat, carbohydrate, and protein) [27], with very little attention to eating behaviors. Considering the high prevalence of CVD risk factors in the police force and the strong associations between emotional eating and CVD risk factors, understanding emotional eating and its association with CVD risk factors in the police force will help guide future strategies to reduce CVD risk factors in NC law enforcement officers. Thus, the purpose of this study was to explore the relationships between emotional eating in response to various emotions (e.g., anger, depression, and anxiety) and CVD risk factors (i.e., body weight, body mass index, waist and hip circumference, waist-to-hip ratio, systolic and diastolic blood pressure, mean arterial pressure, total cholesterol, high- and low-density lipoprotein cholesterol, and glucose levels) among law enforcement officers in NC. We have also considered the differences in emotional eating and CVD risk factors based on sex. Because men and women may have different responses to emotional eating, and the CVD risk factors also vary between them [28,29], it is important to examine the sex variable. This will help identify any potential disparities that can influence the association between emotional eating and CVD risk factors.

## 2. Materials and Methods

### 2.1. Study Design and Setting

The Carolina Blue Project investigates the frequency and severity of disordered eating and its association with CVD risk factors in NC police force using a cross-sectional design. NC has 504 law enforcement agencies employing 23,442 sworn law enforcement officers [30] NC has 100 counties, including 6 urban counties (i.e., an average population density that exceeds 750 people per square mile, including Durham, Forsyth, Guilford, Mecklenburg, New Hanover, and Wake), 16 suburban counties (i.e., an average population density between 250 and 750 people per square mile), and 78 rural counties (i.e., an average population density of 250 people per square mile or less) [31]. Our data collection was conducted from 1 February to 14 November 2023.

### 2.2. Participants

Participants’ inclusion criteria were as follows: (1) sworn police officers, police cadet, deputy sheriffs, state highway patrol troopers, probation, or parole officers, correctional or detention officers, school officers, park rangers, wildlife officers, or any active-duty law enforcement officer who work in NC and (2) age ≥ 18 years. Due to the potential increased risk of bleeding for blood sample collection, officers with coagulation disorders were excluded from the study.

### 2.3. Procedure

All participants gave their informed consent for inclusion before they participated in the study. The study was conducted in accordance with the Declaration of Helsinki, and the protocol was approved by the Ethics Committee of the University of North Carolina at Chapel Hill (approval no. 22-2052). To facilitate stakeholder involvement, we utilized a five-step engagement strategy [32], consisting of the following: (1) identifying key stakeholders within the local law enforcement departments involved in decision-making processes; (2) presenting the need to understand disordered eating behaviors and CVD risk factors of NC law enforcement officers to stakeholders; (3) discussing the project’s activities and collaborative details with stakeholders, including on-site data collection; (4) collaborating resolution of potential issues; and (5) embedding engagement efforts.

The recruitment and data collection process are as follows: (1) On-site recruitment: Law enforcement officers who were interested in participating in the study requested a group health assessment day at their workplace to complete in-person health assessments for a group of law enforcement officers on-site. During the assessment day, participants were screened for eligibility. Eligible participants were then invited to review the consent form and ask questions before being requested to provide consent in a private room. The purpose of the study, procedures, and potential benefits and harms were fully explained to the eligible participants. Informed consent forms were obtained before any health assessment. After the health assessments were completed, participants were instructed to complete an online survey via REDCap [33]. The REDCap system is provided by the University of North Carolina at Chapel Hill and ensures a secure way to administer online questionnaires through an online website [33]. Data were stored in a secure, password-protected file located on a secure server at the University of North Carolina at Chapel Hill. No questionnaires on REDCap contain any questions about participants’ personally identifiable information, such as name, birthday, social security number, phone number, or name of the work department. Each participant was assigned a unique participant identification number on REDCap. Study flyers and brochures containing a quick response (QR) code for officers to access the REDCap survey were also placed at local law enforcement departments. (2) Online recruitment: Law enforcement officers accessed the online survey first through the project website, Facebook advertisement, and the QR code on flyers or brochures. The online survey contained three screening questions: (a) Are you at least 18 years old? (b) Are you a current North Carolina law enforcement personnel? (c) Do you have a history of coagulation disorders? If participants answered “yes” to the first two screening questions and “no” to the third screening question, they were directed to read and sign an online informed consent for the online survey. Potential participants emailed the study Principal Investigator to discuss any questions before providing consent. After informed consent was obtained, participants were directed to complete additional surveys. After all the surveys were completed, participants were asked if they were interested in completing the in-person health assessment. If not, the study activities ended. If yes, participants were contacted by email to schedule an in-person health assessment at a Biobehavioral Laboratory or their workplace. The second informed consent form was obtained at the beginning of the health assessment session. A $100 U.S. dollar gift card was provided to participants after completing all study activities.

### 2.4. Measurement

#### 2.4.1. Demographics Variables and Health History

Participants were asked to report their age, biological sex, race, ethnicity, job categories, and work county. Participants were also asked if they have or have ever had health histories of diabetes (yes/no), use of high blood pressure medicines (yes/no), or use of high cholesterol medicines (yes/no).

#### 2.4.2. Anthropometric Measurements

The participants’ height and weight were measured. All participants were dressed in lightweight indoor clothes without shoes. Their weight was measured twice with a portable digital scale and was averaged and recorded to the nearest 0.1 kg (kg). Their height was measured twice using a portable Martin stadiometer and was averaged and recorded to the nearest 0.5 cm. BMI was computed as weight in kilograms divided by height in meters squared. Waist circumference (in cm) was measured in cm in a horizontal plane around the abdomen at the level of the iliac crest. Hip circumference (in cm) was measured around the widest portion of the buttocks, with the tape parallel to the floor. Waist-to hip ratio was computed as waist circumference divided by hip circumference. Participants were asked to rest for 5 min, and then blood pressure was taken; they were placed in a sitting position with feet on the floor without crossing their legs, and the back against the back of the chair during the blood pressure measurement. All blood pressure was measured from the left arm unless there was a medical reason not to do so.

#### 2.4.3. Biochemical Measurements

All participants were asked to fast for at least 8 h before biomarker data collection. A fasting whole blood sample (40 μL) was collected from a finger using a fingerstick with a capillary tube within 10 s. The blood sample was immediately dispensed into a Lipid Profile•GLU cassette and analyzed using the Cholestech LDX™ System (Abbott, Orlando, FL, USA) for the blood panel levels [34], including total cholesterol, triglycerides, HDL, low-density lipoprotein cholesterol (LDL), and glucose levels. The Cholestech LDX™ System is a small, portable analyzer and test cassette system [34]. The Lipid Profile•GLU cassette is used for the quantitative determination of total cholesterol, HDL, triglycerides, and glucose in whole blood. The Cholestech LDX System combines enzymatic methodology [35] and solid-phase technology to measure total cholesterol, HDL, triglycerides, and glucose. The cassette separates the plasma from the blood cells. A portion of the plasma flows to the right side of the cassette and is transferred to both the total cholesterol and triglyceride reaction pads. Simultaneously, plasma flows to the left side of the cassette, where the LDL and very low-density lipoproteins are precipitated with dextran sulfate (50,000 MW) and magnesium acetate precipitating reagent [36]. The filtrate, containing both glucose and HDL, is transferred to both the glucose and HDL reaction pads. The Cholestech LDX Analyzer measures total cholesterol (range of values tested: 100–500 mg/dL) and HDL (range of values tested: 0–100 mg/dL) using an enzymatic method based on the method formulation of Allain [37] and Roeschlau [38]. Triglycerides (range of values tested: 40–650 mg/dL) were measured through an enzymatic method based on the hydrolysis of triglycerides by lipase to glycerol and free fatty acids. Glucose (range of values tested: 25–575 mg/dL) was measured using an enzymatic method that employed glucose oxidase to catalyze the oxidation of glucose to gluconolactone and hydrogen peroxide. The Cholestech LDX Analyzer calculated estimated LDL (mg/dL) using the measured values with software version V3.0 and higher [34]. Once the analysis was completed, the cassette and the 40 μL blood sample inside the cassette were discarded. The Cholestech LDX™ System with capillary fingerstick whole blood demonstrated good accuracy and precision compared to venous heparinized whole blood, plasma, and serum samples in controlled laboratory conditions [39].

#### 2.4.4. Emotional Eating

The participants’ emotional eating was measured using the Emotional Eating Scale [26] The original Emotional Eating Scale is a 25-item self-report measure with three subscales to measure the urge to eat in response to Anger/Frustration (11 items), Depression (5 items), and Anxiety (9 items) [26]. Items are scored on a 5-point Likert-type scale that assesses the urge to eat in response to each emotion (i.e., 1 = no desire to eat, 2 = a small desire to eat, 3 = a moderate desire to eat, 4 = a strong urge to eat, and 5 = an overwhelming urge to eat). Higher sum scores indicate a greater urge to eat in response to emotions [26]. The original Emotional Eating Scale has been used in women with obesity with the Cronbach’s alpha of 0.81 for the total scale and 0.78, 0.72, and 0.78 for Anger/Frustration, Depression, and Anxiety subscales, respectively [26]. The scale has demonstrated good construct, criterion, and discriminant validity in women with obesity [26]. Goldbacher and colleagues examined the factor structure of the Emotional Eating Scale with the original 25 items in women and men with overweight and obesity and indicated a four-factor structure of Anger (6 items), Depression (9 items), Anxiety (4 items), and Somatic Arousal (6 items) [40]. Somatic arousal involves physical symptoms of heightened physiological arousal that occur in response to stress, trauma, or intense emotional experiences, such as the state of being jittery, shaky, or uneasy [40]. The Cronbach’s alpha of the four-factor structure of the Emotional Eating Scale in women and men with overweight and obesity was 0.94 for the total scale and 0.85, 0.89, 0.78, and 0.79 for Anger, Depression, Anxiety, and Somatic Arousal subscales, respectively [40]. The four-factor structure of the Emotional Eating Scale was used for the current study. The Cronbach’s alpha was 0.97 for the total scale and 0.92, 0.95, 0.90, and 0.84 for Anger, Depression, Anxiety, and Somatic Arousal subscales, respectively, in the current study.

### 2.5. Statistical Analysis

Descriptive statistics (mean and standard deviation or frequency and percentage, as appropriate) were computed for demographic variables, health histories, body weight and height, BMI, waist circumference, hip circumference, waist-to-hip ratio, blood pressure, lipid and glucose biomarkers, and scores of the four emotional eating subscales. We classified job categories according to the job titles provided by the participants as follows: (1) Police Officer, including Police Officer, Inspector, Investigator, Sergeant, Detective, Captain, Lieutenant, K9 handler, Major, Park Ranger, Wildlife Officer, Public Safety Officer, School Officers, Police Cadet, and Community Resource Officer, (2) Deputy Sheriff and Trooper, and (3) Other, including Probation and Parole Officer, Correctional Officers, Detention Officers, and Transportation Officer. Furthermore, participants’ work counties were classified as urban county, suburban county, or rural county. Independent sample t-tests were performed to compare emotional eating and CVD risk factors in male and female participants. Pearson’s correlation analysis was used to determine the bivariate relationships between pairs of all continuous variables. Thirteen multiple linear regression models were analyzed to determine the associations between emotional eating (as the independent variables, including the emotional eating subscales for anger, depression, anxiety, and somatic) and measures of CVD risk factors (as the dependent variables, including body weight, BMI, waist circumference, hip circumference, waist-to-hip ratio, systolic and diastolic blood pressure, mean arterial pressure, total cholesterol, triglycerides, HDL, LDL, and glucose levels), controlling for covariates. We adjusted for the covariate of age for all models because previous research suggests that younger adults tend to engage in emotional eating more frequently than older adults [41] and age was significantly correlated with all variables (all *p* < 0.05) in our sample. In addition, we adjusted for the covariate of using blood pressure medicine when analyzing systolic blood pressure, diastolic blood pressure, and mean arterial pressure as dependent variables, the covariate of using cholesterol medicine when analyzing total cholesterol, triglycerides, HDL, and LDL levels as dependent variables, and the covariate of diabetes history when analyzing glucose level as a dependent variable. The multiple linear regression models were performed on the total sample, as well as separately for males and females. Multiple linear regression coefficient estimates (β), confidence intervals (95% CIs), and *p*-values were noted. For regression model diagnostics, histograms were generated to evaluate whether variables were normally distributed and plots of the residuals versus predicted values were generated to assess the assumption of homogeneity of variances for all models. The result of the diagnostics showed that the assumptions of normality and homogeneity of variance were not violated. The percentage of missing data ranges from 0.5% to 53% across all the variables. All variables of CVD risk factors have more than 5% missing. All missing data were imputed using multiple imputation before the analysis of Pearson’s correlations and multiple linear regression [42]. Because we considered this an exploratory study, we chose a priori not to correct for multiple testing in our correlation analysis and regression models. All data were analyzed using SAS 9.3 software [43] *p*-values less than 0.05 were considered statistically significant.

### 2.6. Geographical Graph

**Figure 1:**
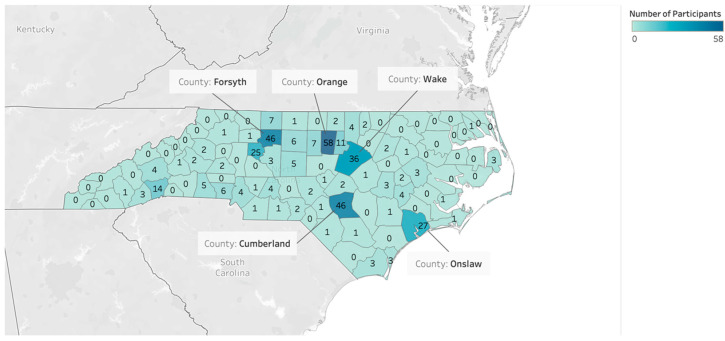
The number of participants included by North Carolina county.

Tableau Desktop (version 2020.3) was utilized to create the geographical graph (Figure 1) that showcased the number of officers participating in the study from each NC County [44]. Tableau Desktop provides the means to not save the visualizations to a public server rather the local computer server [44]. The dataset was input to the software, where the NC Counties’ names were utilized to generate coordinates (latitude, longitude) that could be used to create the figure. The coordinate points and number of participant data work together to produce the figure, and the top five counties with the highest number of participants are labeled.

## 3. Results

### 3.1. Participant Characteristics and Descriptive Statistics Results

During the eligibility screening, 609 participants expressed interest in participating. Of these, 13 participants were ineligible, 159 eligible participants did not respond to any of the survey items (100% missing data) and were excluded, and 32 participants were excluded due to repeat enrollment. Consequently, complete survey data were obtained from 405 participants, and 221 of them completed the health assessment. Figure 1 shows the number of participants based on NC counties. In total, 56 counties participated in the Carolina Blue Project. The number of participants varies from 1 to 58 across different countries. The top five participating counties were Orange, Forsyth, Cumberland, Wake, and Onslow, which are urban and suburban counties.

Table 1 presents the results of the participant characteristics and the descriptive statistics of all measures. The study sample included 405 officers (267 men, 136 women, 2 missing data) who had a mean (±standard deviation) age of 38.12 ± 9.67 years and a mean BMI of 31.45 ± 6.18 kg/m^2^. The majority of participants self-identified as Non-Hispanic White (79.55%) police officers (63.77%) working in suburban counties (45.41%) with no history of diabetes (90.80%) or use of blood pressure (78.81%) or cholesterol medication (87.72%). For other anthropometric and biochemical measures, the results show that the mean BMI is 31.45 ± 6.18 kg/m^2^, and over 50% of participants had BMI exceeding 30 kg/m^2^ (healthy BMI range: 18.5–24.9 kg/m^2^; overweight: BMI 25–29.9 kg/m^2^; obese: BMI ≥ 30 kg/m^2^) [45]. The mean waist circumference for total sample is 103.08 ± 15.88 cm (male officers: 107.08 ± 15.19 cm, female officers: 96.57 ± 15.01 cm, healthy range: less than or equal to 94 cm for male and less than or equal to 80 cm for female) [45], and the mean waist-to-hip ratio for total sample is 0.93 ± 0.37 (male officers: 0.97 ± 0.46, female officers: 0.86 ± 0.08, healthy range: below 0.9 for male and below 0.8 for female) [45]. The mean systolic (125.34 ± 16.37 mmHg) and diastolic (84.11 ± 11.49 mmHg) blood pressures were slightly higher than the desirable level (i.e., 120/80 mmHg) [46]. Over 50% of the participants had a total cholesterol level exceeding 170 mg/dL. About 26% of participants had a triglyceride level greater than 150 mg/dL. About 67% of participants had an HDL level less than 50 mg/dL. The mean LDL (107.37 ± 34.11 mg/dL) was also slightly higher than the desirable level (i.e., less than 100 mg/dL) [47], and almost 60% of the participants had an LDL level greater than 100 mg/dL. About 12% of participants had a glucose level greater than 100 mg/dL.

### 3.2. Differences in Emotional Eating and CVD Risk Factors between Sex Groups

Table 2 presents the results of comparing emotional eating and CVD risk factors based on sex groups. Male participants demonstrated higher mean body weight, waist circumference, systolic blood pressure, diastolic blood pressure, mean arterial pressure, LDL level, and lower HDL level than their female counterparts. The effect size ranges from 0.51 to 1.02.

### 3.3. Correlation Analysis Results

Table 3 displays the correlation analysis results for continuous variables. For the anthropometric measures, emotional eating in response to anger, depression, anxiety, and somatic arousal was significantly positively correlated with body weight, BMI, waist and hip circumferences, and waist-to-hip ratio. For blood pressure measures, only emotional eating in response to somatic arousal was significantly positively correlated with systolic blood pressure. Emotional eating in response to depression and anxiety was significantly negatively correlated with diastolic blood pressure and mean arterial pressure. For the biochemical measurements, emotional eating in response to anger, depression, anxiety, and somatic arousal was significantly negatively correlated with total cholesterol, triglycerides, LDL, and glucose levels. The HDL level was significantly negatively correlated with emotional eating in response to anger but significantly positively correlated with emotional eating in response to somatic arousal.

### 3.4. Relationship between Emotional Eating and CVD Risk Factors

Table 4 shows the multiple linear regression analysis results for the association between emotional eating and cardiovascular risk factors. In Model 1, the results show a significant positive association between emotional eating in response to anger and body weight after adjusting for age (*β* = 1.51, *t* = 2.07, *p* = 0.04), indicating that when emotional eating in response to anger increases by one unit, the mean body weight increases by 1.51 kg. In Models 7 and 8, the results show positive associations between emotional eating in response to anger with both diastolic blood pressure (*β* = 0.83, *t* = 2.18, *p* = 0.03) and mean arterial pressure (*β* = 0.84, *t* = 2.19, *p* = 0.03) after adjusting for age and use of blood pressure medicine, indicating that when emotional eating in response to anger increases by one unit, the mean diastolic blood pressure increases by 0.83 mmHg and the mean arterial pressure increases by 0.84 mmHg. In Model 10, emotional eating in response to depression was significantly positively associated with triglyceride level (*β* = 5.28, *t* = 2.49, *p* = 0.02) after adjusting for age and use of cholesterol medicine, indicating that when emotional eating in response to depression increases by one unit, the mean triglyceride level increases by 5.28 mg/dL. But the emotional eating in response to anxiety was significantly negatively associated with triglyceride level (*β* = −11.42, *t* = −2.64, *p* = 0.01) after adjusting for age and use of cholesterol medicine, indicating that when emotional eating in response to anxiety increases by one unit, the mean triglyceride level decreases by 11.42 mg/dL. No statistically significant results were found between emotional eating in response to somatic arousal and CVD risk factors.

Table 5 and Table 6 show the multiple linear regression analysis results for the association between emotional eating and cardiovascular risk factors in male and female. In male participants, the results show a significant positive association between emotional eating in response to depression and triglycerides (*β* = 6.46, *t* = 2.56, *p* = 0.01) after adjusting for age and use of cholesterol medicine, indicating that when emotional eating in response to depression increases by one unit, the mean triglyceride level increases by 6.46 mg/dL. However, emotional eating in response to anxiety was significantly negatively associated with triglyceride level (*β* = −12.47, *t* = −2.41, *p* = 0.01) after adjusting for age and use of cholesterol medicine, indicating that when emotional eating in response to anxiety increases by one unit, the mean triglyceride level decreases by 12.47 mg/dL. No statistically significant results were found between emotional eating and CVD risk factors in female participants.

## 4. Discussion

Our study indicates that emotional eating in response to anger, depression, anxiety, and somatic arousal were significantly correlated with various anthropometric measures, blood pressure, and CVD-related biochemical markers in NC law enforcement officers. Emotional eating in response to anger was found to be associated with increased body weight, diastolic blood pressure, and mean arterial pressure among the participants. However, after separating male and female participants in our analysis, these associations were no longer significant. Emotional eating in response to depression was associated with higher levels of triglycerides, while anxiety-related emotional eating showed a negative association. This was observed in both the total sample and male participants, but not female participants. Overall, our results highlight the impact of emotional eating on CVD risk factors in NC law enforcement officers.

Our findings showed that higher emotional eating in response to anger was associated with higher body weight. This finding is consistent with previous emotional eating studies on U.S. military personnel and the general population [19,48]. Higher self-reported frequency of emotional eating was associated with higher BMI among 1451 soldiers in the U.S. Army (*β* = 0.65, *p* = 0.004) [19]. Higher levels of emotional eating were associated with an increase in BMI over time (*β* = 0.18, *p* = 0.004), according to a study conducted on a large adult population in the Netherlands (*n* = 1562) [48]. A plausible explanation accounting for the observed association is that individuals may engage in emotional eating as an adaptive response to mitigate the impact of stress or negative emotional states, thereby potentially contributing to subsequent weight gain [49,50]. For example, emotional eating partially mediated the association between stress and body fat percentage in U.S. military personnel [20] indicating that stress or negative emotions may lead to a cycle of overeating as individuals attempt to cope with or suppress their negative feelings through eating [51]. Over time, this can contribute to a pattern of unhealthy eating habits and weight gain [51]. Furthermore, emotional eating often involves the consumption of high-calorie, comfort foods that are typically rich in sugars and fats, and these foods can contribute to an excess of calorie intake, leading to weight gain over time [52]. Despite the ongoing debate regarding whether the measures of emotional eating can accurately reflect the amount of excess food an individual consumes [53] evidence suggests that emotional eating is linked to higher BMI, increased body fat, and weight gain [15,54]. It is noteworthy that, within our sample comprising law enforcement officers routinely exposed to stressful and life-threatening situations while experience the anti-police climate in recent years [55], only emotional eating related to anger demonstrated a significant association with body weight. However, caution should be exercised when interpreting this result as our current study lacks a specific measure for anger. Additional studies are warranted to further investigate the relationship between the emotion of anger, overeating, and weight gain.

Our data showed that higher emotional eating related to anger was associated with higher diastolic blood pressure and mean arterial pressure. Our findings are consistent with prior studies that reported higher degree of emotional eating was associated with higher diastolic blood pressure after adjusting for age, NSAIDS, antidepressants, and antihypertensive medicines among women (*β* = 0.29, *p* = 0.04) [56], and emotional eating was associated with higher systolic blood pressure in a cross-sectional study among adults with type 2 diabetes (*β* = 0.24, *p* = 0.04) [57]. It is possible that the high blood pressure we observed may be associated with the emotion of anger. When individuals experience anger, their body’s “fight or flight” response is activated. This results in the release of stress hormones like adrenaline, which constricts blood vessels and increases heart rate to supply more blood to the muscles, preparing the body for physical action [58]. Another possible explanation is that higher emotional eating in response to anger was associated with higher body weight in our sample. Excess body weight is a well-established risk factor for high blood pressure [59]. The additional adipose tissue requires more blood supply, leading to increased demand on the cardiovascular system and contributing to elevated blood pressure [60]. However, our result needs to be interpreted with caution. The Anger subscale of the Emotional Eating Scale only measures the tendency to eat in response to the emotion of anger, including if they feel an urge to eat when experience resentful (item 1), rebellious (item 7), irritated (item 12), jealous (item 13), furious (item 17), and angry (item 21). We did not measure how much food the participants consumed when they were feeling angry. Therefore, our findings can only reflect the association between the urge to eat when experiencing anger and blood pressure levels. In addition, our study did not include a quantitative measure of anger expression (i.e., to hold anger inside without any outlet or express it through verbal or aggressive behaviors) and the association with the urge to eat [61]. It would be valuable for future studies to investigate the association between anger expression, disordered eating behaviors, and CVD risk factors by utilizing measures such as the State-Trait Anger Expression Inventory [62]. Furthermore, the relationships between emotional eating related to anger and blood pressure were no longer significant after separating male (*n* = 267) and female (*n* = 136) participants. It is possible that the small sample size in the current study may have limited its statistical power to identify significant relationships between emotional eating and blood pressure.

Our findings show mixed results in the relationships between emotional eating and triglyceride levels. Specifically, triglyceride level was positively associated with emotional eating in response to depression, but negatively associated with emotional eating in response to anxiety in our total sample and male participants, but not female participants. Our result is inconsistent with a previous study showing no statistically significant association between emotional eating and triglyceride level in 200 adults in Istanbul [63]. It is possible that the difference in results between our study and the study conducted in Istanbul can be attributed to the methodology used to measure emotional eating. While the study in Istanbul used the Dutch Eating Behavior Questionnaire, this questionnaire did not distinguish between different types of emotions that may trigger emotional eating [63]. In addition, the mixed results of the triglyceride levels in our findings may be due to the complex interplay of psychological and physiological factors that influence eating behavior as a result of depression and anxiety. Depression and anxiety are two mental health conditions that can affect a person’s appetite in various ways [64]. While they can exist together, either can cause an increase or decrease in appetite [64], which may ultimately impact triglyceride levels. Although our t-tests results show no statistical significance between male and female’s emotional eating and triglycerides levels, further research is needed to explore the complex relationship between male and female’s emotional eating in response to depression and anxiety, coping mechanisms, biological responses, food consumption, and how it affects triglyceride levels. Understanding these connections can contribute to more targeted interventions and personalized approaches to address the impact of emotional eating on metabolic health.

This study should be considered within the context of its limitations. First, because we considered this an exploratory study, we did not determine a minimum sample size through a power analysis before recruitment and data collection. As a result, only 221 of the 405 officers participated in the health assessment and provided CVD related biomarkers, which limits its statistical power to identify significant relationships between emotional eating and CVD risk factors. For example, we performed post hoc power analyses using G*Power (version 3.1) [65] based on multiple linear regression coefficient estimates as effect sizes (Table 3) to evaluate the power of the multiple linear regression models. For the nonsignificant relationships between emotional eating in response to anxiety and waist-to-hip ratio, the power value was only 29% (effect size = 0.02, *α* = 0.05, *n* = 221) to analyze the relationships. Second, we did not take into account other important factors that may affect CVD risk, such as diet and physical activity, which may limit our understanding of the comprehensive picture of CVD development and progression. Third, we were not able to conclude temporal causality because the independent (i.e., emotional eating) and dependent variables (i.e., CVD risk factors) were simultaneously measured [66]. Fourth, the findings cannot be assumed to be generalizable to the population of NC law enforcement because most participants were Non-Hispanic White and less than 30% of participants came from NC rural counties. Moreover, our findings cannot speak to clinical levels of emotional eating because the Emotional Eating Scale is not a clinical diagnostic tool and can only indicate the urge to eat in response to a variety of negative emotions [40].

## 5. Conclusions

To our knowledge, this is the first study focusing on emotional eating and CVD risk factors among NC law enforcement officers, who are an understudied population in the disordered eating fields of research. The study reveals that a greater tendency toward emotional eating, specifically as a response to anger, is linked to higher body weight and blood pressure. On the other hand, the connection between emotional eating in response to depression and anxiety with triglyceride levels is inconsistent. No significant evidence was found to support a link between emotional eating as a response to somatic arousal symptoms and CVD risk factors. The well-established relationship observed in both past and current studies between emotional eating and body weight or BMI underscores the need for the inclusion of law enforcement personnel in research related to disordered eating and CVD. This inclusion is vital to addressing and mitigating health disparities within this specific population. By bridging this gap, findings from future studies will contribute to a broader understanding of emotional eating and offer essential insights into the health challenges faced by a population crucial to public safety. Our study highlights the importance of improving the well-being of law enforcement officers for the betterment of their overall health and, consequently, the effectiveness of their crucial role in society.

## Figures and Tables

**Table 1:** Descriptive statistics of all variables in total sample.

Variables		*n*	Mean (SD) or Number (%)	
Age		361	38.12	(9.67)
Biological sex				
	Male	267	267	(66.25%)
	Female	136	136	(33.75%)
Race				
	Non-Hispanic White	315	315	(79.55%)
	Non-Hispanic Black	58	58	(14.65%)
	Other ^a^	23	23	(5.80%)
Ethnicity				
	Hispanic	21	21	(5.24%)
	Non-Hispanic	371	371	(92.52%)
	Prefer not to answer	9	9	(2.24%)
Job categories				
	Police officer ^b^	257	257	(63.77%)
	Deputy sheriff and trooper	43	43	(10.67%)
	Other ^c^	103	103	(25.56%)
Work county				
	Urban	104	104	(25.81%)
	Suburban	183	183	(45.41%)
	Rural	116	116	(28.78%)
Diabetes				
	Yes	30	30	(9.20%)
	No	296	296	(90.80%)
Blood pressure medicine use				
	Yes	71	71	(21.19%)
	No	264	264	(78.81%)
Cholesterol medicines use				
	Yes	21	21	(12.28%)
	No	150	150	(87.72%)
Emotional eating: Anger		341	8.30	(4.10)
Emotional eating: Depression		341	15.69	(7.95)
Emotional eating: Anxiety		340	6.52	(3.58)
Emotional eating: Somatic arousal		340	9.60	(4.26)
Body weight (kg)		221	94.22	(23.01)
Body height (cm)		221	172.50	(9.82)
BMI (kg/m^2^)		221	31.45	(6.18)
	19–24 kg/m^2^	27	27	(12.22%)
	25–29 kg/m^2^	76	76	(34.39%)
	30–39 kg/m^2^	98	98	(44.34%)
	≥40 kg/m^2^	20	20	(9.05)
Waist circumference (cm)		221	103.08	(15.88)
Hip circumference (cm)		221	113.21	(13.26)
Waist-to-hip ratio		221	0.93	(0.37)
Systolic blood pressure (mmHg)		221	125.34	(16.37)
Diastolic blood pressure (mmHg)		221	84.11	(11.49)
Mean arterial pressure (mmHg)		221	97.86	(12.32)
Total cholesterol (mg/dL)		215	174.64	(37.17)
	<170 mg/dL	103	103	(47.91%)
	≥170 mg/dL	112	112	(52.09%)
Triglycerides (mg/dL)		202	127.51	(84.86)
	<150 mg/dL	149	149	(73.76%)
	150–199 mg/dL	23	23	(11.39%)
	200–499 mg/dL	28	28	(13.86%)
	≥500 mg/dL	2	2	(0.99%)
HDL (mg/dL)		216	44.73	(14.78)
	<50 mg/dL	145	145	(67.13%)
	≥50 mg/dL	71	71	(32.87%)
LDL (mg/dL)		190	107.37	(34.11)
	<100 mg/dL	78	78	(41.05%)
	100–189 mg/dL	110	110	(57.90%)
	≥190 mg/dL	2	2	(1.05%)
Glucose (mg/dL)		221	92.17	(30.73)
	<99 mg/dL	195	195	(88.24%)
	100–125 mg/dL	15	15	(6.78%)
	>126 mg/dL	11	11	(4.98%)

*n* = number of participants; BMI = body mass index; HDL = high-density lipoprotein cholesterol; LDL = low-density lipoprotein cholesterol; kg = kilogram; cm = centimeter; ^a^ = including American Indian, Native Alaskan, Asian, and mixed race; ^b^ = including Police Officer, Inspector, Investigator, Sergeant, Detective, Captain, Lieutenant, K9 handler, Major, Park Ranger, Wildlife Officer, Public Safety Officer, School Officers, Police Cadet, and Community Resource Officer; ^c^ = including Probation and Parole Officer, Correctional Officers, Detention Officers, and Transportation Officer; Urban = an average population density that exceeds 750 people per square mile; Suburban = an average population density between 250 and 750 people per square mile; Rural = an average population density of 250 people per square mile or less.

**Table 2:** Differences in emotional eating and CVD risk factors between sex groups.

	Male (*n* = 267)		Female (*n* = 136)				
Variables	Mean	SD	Mean	SD	*t*	*p*	Cohen’s d
Emotional eating: Anger	8.36	4.19	8.19	3.95	0.35	0.72	0.04
Emotional eating: Depression	15.59	8.09	15.91	7.73	−0.34	0.73	−0.04
Emotional eating: Anxiety	6.56	3.64	6.47	3.49	0.22	0.83	0.03
Emotional eating: Somatic arousal	9.59	4.37	9.64	4.10	−0.10	0.93	−0.01
Body weight (kg)	101.9	22.60	81.84	17.66	7.33	<0.01	1.02
BMI (kg/m^2^)	32.06	6.12	30.49	6.23	1.84	0.07	0.26
Waist circumference (cm)	107.1	15.19	96.63	14.93	4.98	<0.01	0.69
Hip circumference (cm)	113.9	13.69	112.2	12.53	0.93	0.35	0.13
Wasit-to-hip ratio	0.97	0.46	0.86	0.08	2.74	0.01	0.38
Systolic blood pressure (mmHg)	129.8	13.96	117.9	17.49	5.29	<0.01	0.74
Diastolic blood pressure (mmHg)	86.36	10.59	80.53	12.07	3.75	<0.01	0.52
Mean arterial pressure (mmHg)	100.9	10.92	92.98	13.04	4.81	<0.01	0.67
Total cholesterol (mg/dL)	177.7	39.97	169.9	31.74	1.57	0.12	0.22
Triglycerides (mg/dL)	128.7	87.34	125.0	81.61	0.30	0.77	0.04
HDL (mg/dL)	40.28	12.88	52.00	14.87	−6.12	<0.01	−0.86
LDL (mg/dL)	113.9	34.83	96.85	30.30	3.45	<0.01	0.51
Glucose (mg/dL)	95.54	33.32	86.88	25.26	2.18	0.03	0.30

BMI = Body Mass Index; HDL = high-density lipoprotein cholesterol; LDL = low-density lipoprotein cholesterol; kg = kilogram; cm = centimeter; SD = standard deviation. *t* = *t* value from the independent sample *t* tests.

**Table 3:** Correlation between emotional eating, cardiovascular disease risk factors, and age in total sample.

	Emotional Eating: Anger	Emotional Eating: Depression	Emotional Eating: Anxiety	Emotional Eating: Somatic Arousal
	*r*	*r*	*r*	*r*
Body weight (kg)	0.21 **	0.15 **	0.17 **	0.14 **
BMI (kg/m^2^)	0.18 **	0.16 **	0.18 **	0.16 **
Waist circumference (cm)	0.19 **	0.17 **	0.17 **	0.15 **
Hip circumference (cm)	0.11 **	0.07 **	0.10 **	0.04 *
Waist-to-hip ratio	0.02 **	0.08 **	0.03	0.08 **
Systolic blood pressure	0.06	−0.02	0.01	0.06 **
Diastolic blood pressure	−0.01	−0.06 **	−0.08 **	−0.05
Mean arterial pressure	0.02	−0.05 **	−0.05 **	−0.004
Total cholesterol (mg/dL)	−0.05 **	−0.06 **	−0.09 **	−0.02
Triglycerides (mg/dL)	−0.05 **	0.01	−0.08 **	−0.07 **
HDL (mg/dL)	−0.03 *	−0.02	−0.01	0.03 *
LDL (mg/dL)	−0.01	−0.06 **	−0.06 **	−0.01
Glucose (mg/dL)	−0.06 **	0.003	−0.03	−0.07
Age	0.07 **	0.07 **	0.07 **	0.07 **

BMI = body mass index; *r* = Pearson’s correlation coefficient; * *p* < 0.05; ** *p* < 0.01.

**Table 4:** Multiple linear regression results for associations of emotional eating and cardiovascular disease risk factors in total sample.

	Emotional Eating: Anger		Emotional Eating: Depression		Emotional Eating: Anxiety		Emotional Eating: Somatic Arousal	
Dependent Variables	*β*	95% CI	*β*	95% CI	*β*	95% CI	*β*	95% CI
1. Body weight (kg)	1.51 *	(0.06, 2.96)	−0.13	(−1.00, 0.75)	0.14	(−2.15, 2.44)	−0.32	(−1.78, 1.15)
2. BMI (kg/m^2^)	0.19	(−0.23, 0.62)	−0.01	(−0.22, 0.19)	0.12	(−0.40, 0.65)	0.02	(−0.38, 0.41)
3. Waist circumference (cm)	0.71	(−0.53, 1.96)	0.15	(−0.38, 0.67)	−0.13	(−1.67, 1.42)	−0.14	(−1.11, 0.83)
4. Hip circumference (cm)	0.73	(−0.22, 1.67)	−0.20	(−0.72, 0.32)	0.50	(−1.00, 2.00)	−0.50	(−1.32, 0.33)
5. Wasit-to-hip ratio	−0.02	(−0.04, 0.01)	0.01	(−0.01, 0.03)	−0.02	(−0.06, 0.01)	0.01	(−0.01, 0.03)
6. Systolic blood pressure	0.85	(−0.15, 1.86)	−0.45	(−1.03, 0.13)	−0.50	(−1.87, 0.87)	0.37	(−0.44, 1.18)
7. Diastolic blood pressure	0.83 *	(0.07, 1.60)	−0.04	(−0.54, 0.46)	−0.91	(−1.86, 0.05)	−0.27	(−0.91, 0.36)
8. Mean arterial pressure	0.84 *	(0.08, 1.61)	−0.18	(−0.65, 0.29)	−0.77	(−1.77, 0.24)	−0.06	(−0.71, 0.59)
9. Total cholesterol (mg/dL)	0.04	(−3.18, 3.26)	0.45	(−1.55, 2.45)	−2.92	(−7.25, 1.41)	0.94	(−1.42, 3.30)
10. Triglycerides (mg/dL)	0.68	(−8.31, 9.67)	5.28 *	(0.99, 9.58)	−11.42 **	(−20.02, −2.83)	−2.71	(−8.89, 3.46)
11. HDL (mg/dL)	−0.82	(−2.18, 0.55)	−0.14	(−0.72, 0.44)	0.41	(−1.06, 1.89)	0.75	(−0.08, 1.59)
12. LDL (mg/dL)	0.73	(−2.21, 3.68)	−0.42	(−2.45, 1.61)	−1.11	(−5.58, 3.36)	0.67	(−1.35, 2.69)
13. Glucose (mg/dL)	−1.27	(−3.65, 1.10)	0.68	(−0.37, 1.72)	−0.46	(−2.91, 1.98)	−0.56	(−2.05, 0.94)

BMI = body mass index; HDL = high-density lipoprotein cholesterol; LDL = low-density lipoprotein cholesterol; kg = kilogram; m = meter; cm = centimeter; *β* = standardized parameter estimates; 95% CI = 95% confidence interval; Models 1 to 5 were adjusted for age; Models 6 to 8 were adjusted for age and use of blood pressure medicine. Models 9 to 12 were adjusted for age and use of cholesterol medicine; Model 13 was adjusted for age and history of diabetes. * *p* < 0.05; ** *p* < 0.01.

**Table 5:** Multiple linear regression results for associations of emotional eating and cardiovascular disease risk factors in males.

	Emotional Eating: Anger		Emotional Eating: Depression		Emotional Eating: Anxiety		Emotional Eating: Somatic Arousal	
Dependent Variables	*β*	95% CI	*β*	95% CI	*β*	95% CI	*β*	95% CI
1. Body weight (kg)	1.67	(−0.18, 3.52)	0.05	(−0.95, 1.04)	−0.21	(−2.88, 2.45)	−0.26	(−2.07, 1.55)
2. BMI (kg/m^2^)	0.26	(−0.27, 0.78)	−0.01	(−0.26, 0.25)	0.06	(−0.58, 0.70)	0.06	(−0.45, 0.56)
3. Waist circumference (cm)	0.91	(−0.55, 2.38)	0.27	(−0.36, 0.90)	−0.46	(−2.21, 1.28)	−0.07	(−1.26, 1.21)
4. Hip circumference (cm)	0.76	(−0.40, 1.91)	−0.29	(−0.95, 0.36)	0.51	(−1.30, 2.32)	−0.30	(−1.50, 0.60)
5. Wasit-to-hip ratio	−0.02	(−0.05, 0.02)	0.02	(−0.01, 0.04)	−0.03	(−0.08, 0.02)	0.01	(−0.02, 0.04)
6. Systolic blood pressure	1.04	(−0.26, 2.34)	−0.17	(−0.81, 0.47)	−1.02	(−2.72, 0.68)	0.25	(−0.98, 1.48)
7. Diastolic blood pressure	0.89	(−0.21, 1.99)	0.17	(−0.38, 0.72)	−1.33	(−2.52, 0.13)	−0.43	(−1.40, 0.54)
8. Mean arterial pressure	0.94	(−0.09, 1.98)	0.06	(−0.44, 0.57)	−1.23	(−2.47, 0.02)	−0.20	(−1.21, 0.80)
9. Total cholesterol (mg/dL)	−0.22	(−4.45, 4.01)	−0.01	(−2.16, 2.13)	−3.58	(−8.84, 1.68)	2.16	(−1.05, 5.38)
10. Triglycerides (mg/dL)	1.10	(−8.78, 10.99)	6.46 *	(1.44, 11.48)	−12.47 *	(−22.67, −2.27)	−4.55	(−8.89, 3.46)
11. HDL (mg/dL)	−0.87	(−2.54, 0.80)	−0.41	(−1.17, 0.35)	0.51	(−1.02, 2.04)	1.16	(−0.08, 2.24)
12. LDL (mg/dL)	0.45	(−3.40, 4.30)	−0.82	(−3.07, 1.43)	−1.67	(−7.12, 3.78)	1.82	(−0.88, 4.53)
13. Glucose (mg/dL)	−1.95	(−5.51, 1.61)	1.14	(−0.03, 2.31)	−0.72	(−3.74, 2.30)	−0.83	(−2.61, 0.96)

BMI = body mass index; HDL = high-density lipoprotein cholesterol; LDL = low-density lipoprotein cholesterol; kg = kilogram; m = meter; cm = centimeter; *β* = standardized parameter estimates; 95% CI = 95% confidence interval; Models 1 to 5 were adjusted for age; Models 6 to 8 were adjusted for age and use of blood pressure medicine. Models 9 to 12 were adjusted for age and use of cholesterol medicine; Model 13 was adjusted for age and history of diabetes. * *p* < 0.05.

**Table 6:** Multiple linear regression results for associations of emotional eating and cardiovascular disease risk factors in females.

	Emotional Eating: Anger		Emotional Eating: Depression		Emotional Eating: Anxiety		Emotional Eating: Somatic Arousal	
Dependent Variables	*β*	95% CI	*β*	95% CI	*β*	95% CI	*β*	95% CI
1. Body weight (kg)	0.52	(−1.53, 2.58)	−0.19	(−1.41, 1.02)	0.92	(−2.34, 4.18)	−0.47	(−2.12, 1.17)
2. BMI (kg/m^2^)	0.04	(−0.57, 0.66)	−0.03	(−0.39, 0.32)	0.33	(−0.63, 1.30)	−0.12	(−0.63, 0.38)
3. Waist circumference (cm)	−0.05	(−1.74, 1.64)	0.02	(−0.89, 0.93)	0.70	(−1.73, 3.12)	−0.37	(−1.60, 0.86)
4. Hip circumference (cm)	0.58	(−0.95, 2.12)	−0.16	(−0.99, 0.68)	0.74	(−1.58, 3.06)	−0.84	(−1.89, 0.21)
5. Wasit-to-hip ratio	−0.02	(−0.05, 0.02)	0.01	(−0.01, 0.02)	−0.01	(−0.05, 0.05)	0.01	(−0.01, 0.04)
6. Systolic blood pressure	0.27	(−1.32, 1.86)	−0.60	(−1.65, 0.45)	0.36	(−1.87, 2.58)	0.23	(−0.85, 1.31)
7. Diastolic blood pressure	0.57	(−0.77, 1.91)	−0.18	(−1.10, 0.74)	−0.22	(−2.18, 1.75)	−0.15	(−1.02, 0.72)
8. Mean arterial pressure	0.47	(−0.85, 1.79)	−0.32	(−1.23, 0.59)	−0.02	(−1.92, 1.87)	−0.02	(−0.90, 0.85)
9. Total cholesterol (mg/dL)	0.07	(−4.11, 4.25)	0.92	(−1.91, 3.75)	−1.83	(−8.17, 4.51)	−0.25	(−3.44, 2.95)
10. Triglycerides (mg/dL)	−1.99	(−13.95, 9.97)	4.45	(−2.14, 11.04)	−7.73	(−22.18, 6.73)	0.30	(−7.68, 8.28)
11. HDL (mg/dL)	−0.41	(−2.22, 0.55)	−0.10	(−0.99, 0.79)	0.60	(−1.78, 2.98)	0.10	(−1.10, 1.30)
12. LDL (mg/dL)	0.87	(−3.10, 4.84)	0.15	(−2.41, 2.72)	−0.92	(−7.00, 5.16)	−0.43	(−3.42, 2.56)
13. Glucose (mg/dL)	−0.29	(−2.89, 2.32)	0.15	(−1.57, 1.88)	−0.42	(−3.89, 3.05)	0.04	(−1.99, 2.07)

BMI = body mass index; HDL = high-density lipoprotein cholesterol; LDL = low-density lipoprotein cholesterol; kg = kilogram; m = meter; cm = centimeter; *β* = standardized parameter estimates; 95% CI = 95% confidence interval; Models 1 to 5 were adjusted for age; Models 6 to 8 were adjusted for age and use of blood pressure medicine. Models 9 to 12 were adjusted for age and use of cholesterol medicine; Model 13 was adjusted for age and history of diabetes.

## Data Availability

The research materials supporting this publication can be accessed by contacting Dr. Ya-Ke Wu.
